# COVID-19 and myocarditis: a review of literature

**DOI:** 10.1186/s43044-022-00260-2

**Published:** 2022-04-05

**Authors:** Mohammed Ali, Haaris A. Shiwani, Mohammed Y. Elfaki, Moaz Hamid, Rebabonye Pharithi, Rene Kamgang, Christian BinounA Egom, Jean Louis Essame Oyono, Emmanuel Eroume-A Egom

**Affiliations:** 1 School of Medicine, The University of Manchester, Stopford Building, 99 Oxford Road, Manchester, M13 9PG UK; 2 Burnley General Hospital, Burnley, UK; 3 School of Medicine, University College Dublin, Dublin, Ireland; 4 Birmingham Midland Eye Centre, Birmingham, UK; 5 St Vincent’s University Hospital, Dublin, Ireland; 6 Faculty of Medicine, University of N’Djamena, N’djamena, Chad; 7 Laboratory of Endocrinology and Radioisotopes, Institute of Medical Research and Medicinal Plants Studies (IMPM), Yaoundé, Cameroon; 8 Institut du Savoir Montfort (ISM), Hôpital Montfort, 713 Montreal Rd, Ottawa, ON K1K 0T2 Canada

**Keywords:** COVID-19, SARS-CoV-2, Myocarditis, Vaccine, Intravenous immunoglobulins

## Abstract

Myocarditis has been discovered to be a significant complication of coronavirus disease 2019 (COVID-19), a condition caused by the severe acute respiratory syndrome coronavirus 2 (SARS-CoV-2) virus. COVID-19 myocarditis seems to have distinct inflammatory characteristics, which make it unique to other viral etiologies. The incidence of COVID-19 myocarditis is still not clear as a wide range of figures have been quoted in the literature; however, it seems that the risk of developing myocarditis increases with more severe infection. Furthermore, the administration of the mRNA COVID-19 vaccine has been associated with the development of myocarditis, particularly after the second dose. COVID-19 myocarditis has a wide variety of presentations, ranging from dyspnea and chest pain to acute heart failure and possibly death. It is important to catch any cases of myocarditis, particularly those presenting with fulminant myocarditis which can be characterized by signs of heart failure and arrythmias. Initial work up for suspected myocarditis should include serial troponins and electrocardiograms. If myocardial damage is detected in these tests, further screening should be carried out. Cardiac magnetic resonance imagining and endomyocardial biopsy are the most useful tests for myocarditis. Treatment for COVID-19 myocarditis is still controversial; however, the use of intravenous immunoglobulins and corticosteroids in combination may be effective, particularly in cases of fulminant myocarditis. Overall, the incidence of COVID-19 myocarditis requires further research, while the use of intravenous immunoglobulins and corticosteroids in conjunction requires large randomized controlled trials to determine their efficacy.

## Background

An outbreak of pneumonia infections originating in December 2019, Wuhan, China, was given the name coronavirus disease 2019 (COVID-19) [1]. The cause was discovered to be a novel virus, the severe acute respiratory syndrome coronavirus 2 (SARS-CoV-2) [1]. The virus rapidly spread across the globe and on March 11, 2020, the World Health Organization declared COVID-19 a global pandemic [2]. As of August 31, 2021, there have been over 200 million confirmed cases of COVID-19 worldwide with over 4.5 million deaths [3].

The spike (S) protein of the SARS-CoV-2 virus is pivotal for its ability to bind and enter host cells [4]. The S protein has two subunits, namely S1 and S2, with S1 allowing for binding to host cells, while S2 carries out the process of fusion between the membranes of the virus and host cell [4]. Angiotensin-converting enzyme (ACE)-2 is the receptor to which the S protein binds [5]. Once binding has occurred, the SARS-CoV-2 virus is able to fuse its membrane with the host cell, allowing it to enter the host cell [6]. The fusion of membranes is mediated by type 2 transmembrane serine protease (TMPRSS2), a cell surface protein which cleaves ACE-2 [7]. Entry into host cells is followed by viral replication and an immune response, causing tissue damage and the clinical manifestations of COVID-19 [4].

COVID-19 typically manifests as pneumonia resulting in symptoms of cough, dyspnea and fever [8]. However, it has since been realized that COVID-19 can cause cardiovascular complications, among these is myocarditis. The disease course of COVID-19 myocarditis can range from mild to severe. If not treated, the myocarditis may progress to life-threatening heart failure and arrythmias; therefore, it is imperative for clinicians to recognize possible cases of COVID-19 myocarditis and treat them accordingly [9–11]. Moreover, it may lead to an increase in ward admissions in a time where hospitals can already be overwhelmed. This literature review aims to discuss the pathophysiology and incidence of COVID-19 myocarditis, along with its presentation, diagnosis and treatment.

## Pathophysiology of viral myocarditis

Myocarditis is described as inflammation of the heart muscle, leading to damage in the absence of ischemia [12, 13]. Viruses have been suggested to be a significant etiology for myocarditis with a wide variety of causative agents including, but not limited to, adenovirus, parvovirus B19, Epstein Barr virus and cytomegalovirus [13–16]. Now, recent evidence suggests the SARS-CoV-2 virus may also be a significant infectious agent for myocarditis. The proposed pathophysiology of viral myocarditis is a combination of direct cell injury and immune-mediated cell death [12]. Early in the development of viral myocarditis high rates of viral replication leads to direct cardiomyocyte injury [17]. The damaged cells, and proteins released from them (such as cardiac myosin), activate toll-like receptors and inflammasomes, leading to the release of pro-inflammatory cytokines [18, 19]. As time progresses, these pro-inflammatory cytokines recruit immune cells, including natural killer cells, macrophages and T-lymphocytes, to the myocardium. These cells are involved in immune-mediated myocyte injury [17]. Moreover, interleukin (IL)-1β and IL-17 cause cardiac remodeling and fibrosis, which eventually leads to dilated cardiomyopathy and heart failure [20, 21]. Myocardial fibrosis leads to a disruption in the conduction system, leading to an increased risk of developing arrythmias [22].

## Proposed mechanisms for COVID-19 myocarditis 

As mentioned previously, the SARS-CoV-2 virus enters human cells by binding to the ACE2 protein. While the ACE2 protein is expressed on epithelial cells (type II alveolar cells) of the respiratory tract, leading to the respiratory manifestations of COVID-19, these proteins can also be found on cardiomyocytes [23–25]. A case study, using endomyocardial biopsy (EMB), revealed the presence of SARS-CoV-2 viral particles in the myocardium of a patient with COVID-19 [26]. Furthermore, autopsies of 20 human heart samples of patients infected with SARS-CoV, a virus related to SARS-CoV2, demonstrated seven hearts contained viral particles, alongside macrophage infiltration [27]. Therefore, it is very possible that the SARS-CoV-2 virus can also infect cardiomyocytes leading to viral myocarditis [28]. An alternative way SARS-CoV-2 can cause myocardial damage is via the infection of endothelial cells in the heart [29]. This theory is supported by the discovery of SARS-CoV-2 in endothelial cells of numerous organs, including the heart, under histology [30, 31].

Some authors have found an increased number of diffusely distributed CD68 + cells in hearts of patients with COVID-19, compared to those with typical myocarditis and control groups [32]. Fox et al. hypothesized the difference in immune cells on histology suggest COVID-19 myocarditis is a distinct inflammatory process separate from typical viral myocarditis [32]. Two theories describing the inflammatory process were proposed. First, SARS-CoV-2 can infect endothelial cells within coronary vessels, leading to the migration of macrophages to these areas, causing the activation of complement and apoptosis [32]. Second, the inflammation can lead to thrombus formation in the coronary vessels leading to ischemic myocardial injury [32].

**Fig. 1:**
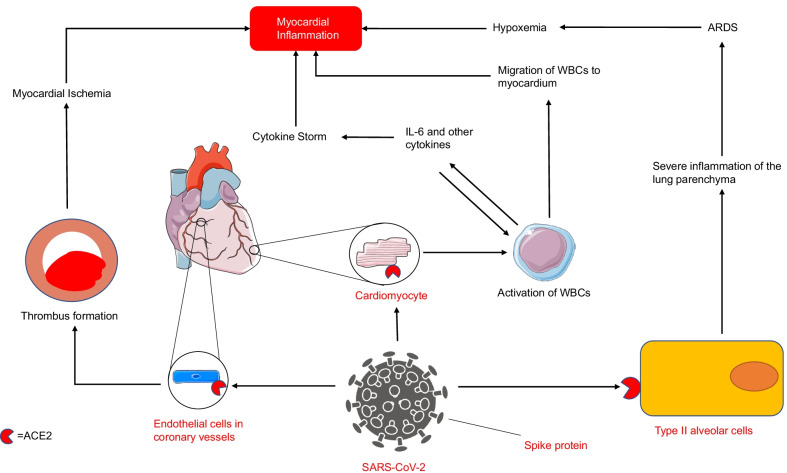
Pathophysiology of COVID-19-induced myocarditis. SARS-CoV-2 uses the Spike protein to bind to and enter a variety of cells including type II alveolar cells, cardiomyocytes and endothelial cells. Myocardial inflammation results from the combination of hypoxemia, thrombus-induced ischemia and the migration of proinflammatory cytokines and cells to the area. ACE2= angiotensin-converting enzyme 2; ARDS= acute respiratory distress syndrome; IL-6= interleukin 6; SARS-CoV-2= severe acute respiratory syndrome coronavirus 2; WBC= white blood cell

Systemic inflammation may also play a role in the development of COVID-19 myocarditis. IL-6 is a cytokine implicated in the pathophysiology of myocarditis, recruiting inflammatory cells to the myocardium [33]. IL-6 is also a primary mediator of the cytokine storm, a life-threatening condition seen in some patients who have developed COVID-19, which is characterized by extreme increases in pro-inflammatory cytokines and an uncontrolled immune response [33, 34]. This systemic inflammation can further increase the risk of thrombus formation within coronary vessels due to activation of platelets and high levels of clotting factors (including factor V and VIII) [29, 35]. It is also possible that the cytokine storm may lead to exacerbation of established myocarditis and further myocardial injury [28]. Furthermore, myocardial injury may be exacerbated by hypoxia of the myocardium due to increased oxygen demands in the setting of infection, which cannot be met due to the presence of pneumonia of acute respiratory distress syndrome [36]. The possible pathophysiology of COVID-19 myocarditis is outlined in Fig. 1.

## Risk of developing COVID-19 myocarditis

Unfortunately, the incidence of COVID-19-induced myocarditis is unclear. A study revealed that approximately 28% of patients with COVID-19 exhibited myocardial injury, diagnosed by the presence of raised troponin T [37]. A meta-analysis found 8% of patients with COVID-19 developed myocardial injury, with a 13-fold increase in prevalence among patients in intensive care [38]. Halushka et al. found 7.2% of 277 postmortem cases displaying evidence of myocarditis, with only less than 2% of cases demonstrating clinically significant myocarditis [39]. This study reveals that the true incidence of COVID-19 myocarditis may be underestimated, as some patients may be asymptomatic or have minor symptoms. Puntmann et al. studied 100 patients who had recently recovered from severe COVID-19 and found 78% exhibited cardiac involvement on cardiac magnetic resonance imaging (cMRI), with 60% found to have ongoing inflammation [40]. Myocarditis is a significant complication of COVID-19; however, the exact mortality due to these complications remains unclear. Published evidence does show that the myocardial involvement increases mortality in patients hospitalized with COVID-19 [41]. Qiurong et al. demonstrated that among 68 deaths of patients with COVID-19, 7% were attributable to fulminant myocarditis leading to circulatory failure, while 33% died of a combination of respiratory and cardiac failure [42]. The diagnoses of fulminant myocarditis were made based on the evaluation of clinical data available to the authors. There was no mention of analysis of immunohistology which would be the most reliable way of diagnosing myocarditis. This may affect reliability of these results due to an increased risk of misdiagnoses. Myocarditis can worsen prognosis for patients who have developed COVID-19 infection, while patients who develop COVID-19 myocarditis may suffer from long-term cardiovascular complications, which will need to be studied over time.

A systematic review and case series demonstrated that patients with cardiovascular comorbidities were more at risk at developing COVID-19 myocarditis [43, 44]. The exact mechanism as to why this occurs is not completely clear. Guo et al. [45] hypothesized that the virus could travel into the pulmonary circulation after infecting pneumocytes via ACE2 expressing endothelial cells. Furthermore, it has been demonstrated that failing hearts show greater expression of ACE2 proteins than normal [45–47]. The higher concentrations of ACE2 in these hearts may allow for easier uptake of the SARS-CoV-2 virus [46]. As the heart is the first organ encountered by pulmonary outflow, the SARS-CoV-2 virus is likely to encounter ACE2 expressing cardiomyocytes in patients with cardiovascular disease [45]. SARS-CoV-2 binds to ACE2 to enter cells meaning these patients may be at a higher risk of developing COVID-19-induced myocarditis [28]. Black, Asian and minority ethnic (BAME) groups may be more seriously affected by COVID-19-induced myocarditis, due to a greater prevalence of cardiovascular disease among these groups [48–52]. However, other evidence suggests that those of African descent express lower levels of ACE2, particularly those of whom with pre-hypertension [52]. The evidence is contradictory and so the association between race and the risk of developing COVID-19 myocarditis requires more research.

An important group to be vigilant of are those who compete in competitive sports as myocarditis is associated with sudden cardiac death in athletes [53]. Daniels et al. tested 1597 athletes for the presence of COVID-19-induced myocarditis. Of these athletes, 37 (2.3%) were diagnosed with COVID-19 myocarditis, 28 of whom were classified as having possible myocarditis [54]. Daniels et al. noted that if cardiac testing were only done on those patients with cardiac symptoms, only five cases of COVID-19 myocarditis would have been recorded [54]. Again, this highlights the possibility that the cardiac involvement of COVID-19 is underestimated due to asymptomatic patients. In another study of 26 competitive athletes who underwent cMRI, 15% were found to have myocarditis, while 31% showed evidence of the previous myocardial damage [55]. Athletes who have recovered from COVID-19 and are returning to sports should receive cardiac testing, including cMRI, to screen for any active myocarditis or the previous cardiac injury.

Overall, the exact incidence of COVID-19 myocarditis is still unclear; however, the current literature suggests that those who suffer from severe infection are at an increased risk of developing myocarditis than those who develop a mild infection.

## Association between COVID-19 mRNA vaccine and myocarditis

The administration of a COVID-19 mRNA vaccine (both Pfizer and Moderna) may be associated with a development of myocarditis. Recent evidence suggest that myocarditis rates are around 12.6 cases per million administrations of the second dose of mRNA vaccines among people aged 12–39, with a predominance of young males [56]. Patients tend to present with chest pain and abnormal ECG findings which occur two to three days after the second dose of the vaccine [56]. Patients usually have their symptoms resolved [56]. Diaz et al. discovered an increase mean number of monthly cases of myocarditis/myopericarditis of 10.4 (p < 0.001) since the vaccine started to be rolled out in the USA [57]. Furthermore, multiple case series following patients who develop acute myocarditis following COVID-19 mRNA vaccination have been published [58–61]. All vaccinated patients studied were confirmed to not have COVID-19 at presentation. Mouch et al. [58] followed five patients presenting after the second dose and one patient presenting after the first dose. Kim et al. [59] followed four patients, all of whom had both doses of the mRNA COVID-19 vaccine. Shaw et al. [60] followed four patients of which two received both doses and two received one dose of the vaccine. Both the patients presenting after the first dose had previous SARS-CoV-2 infection. All patients in these studies underwent diagnostic cMRI. Finally, Montgomery et al. [61] followed 23 male patients, 20 of which presenting following the second dose, while three presented after the first dose (all of whom had previous SARS-CoV-2 infection). However, in this study, only eight patients underwent diagnostic cMRI, affecting the reliability of the results as most diagnoses would have been made by clinical judgment. Overall, these four studies followed 37 patients: 31 presenting after the second dose and 6 presenting after the first dose of the mRNA vaccine with previous COVID-19 infection. This highlights the important point that vaccine-induced myocarditis tends to occur after sensitization to SARS-CoV-2.

A case series following 15 children (12–18 years old) who were hospitalized due to symptoms of myocarditis (such as chest pain and fever) found 13 of these patients had cMRI changes consistent with myocardial inflammation [62]. All but one of these patients presented after the second dose of the vaccine. This study reveals that children may also be at risk of developing myocarditis from administration of mRNA vaccines. Current evidence demonstrates that children with COVID-19 have low mortality and intensive care admission rate [63]. Therefore, recommendations for giving mRNA vaccines to children (under 18 s) should consider the risk of developing myocarditis compared to the risk of severe COVID-19 infection in this age group.

There is evidence that the COVID-19 mRNA vaccine can cause myocarditis, particularly in cases of the previous exposure. With most patients presenting after both doses of the vaccine, or after the first dose with prior SARS-CoV-2 infection, there may be a possibility of a hypersensitivity reaction occurring after the previous exposure [61, 64]. This reaction may be a delayed-type hypersensitivity reaction due to the two- to three-day period between vaccine administration and onset of symptoms seen in most patients [56, 64]. The first vaccine dose could act to sensitize the immune system, while the second dose causes the activation of the effector phase of the immune system [64]. Activated immune cells may migrate to the myocardium leading to release of cytokines into the myocardium. This process can stimulate further immune cells to enter the myocardium, leading to inflammation and the patient may present with myocarditis.

## Presentation of COVID-19 myocarditis

Classic presentation of myocarditis is analogous to heart failure, with symptoms of dyspnea, orthopnea and chest pain maybe present [65]. However, clinical presentations of patients with COVID-19 myocarditis can vary from patient to patient. Some patients have relatively mild presentations such as cough, fever and dyspnea [9, 10, 22, 66, 67]. These symptoms may be due to COVID-19 itself and not the myocarditis. Therefore, some patients may have a silent presentation of COVID-19 myocarditis [22]. Some patients may present with chest pain which may or may not be described as a pressure [68–70]. In one report this chest pain was present without fatigue, cough or dyspnea [68]. Some patients may also present with palpitations alongside their other symptoms [67, 71]. After initial presentation, patients may deteriorate to develop signs of heart failure and hemodynamic compromise if treatment is not initiated or is inadequate [9–11]. In severe cases, patients may initially present with new-onset heart failure in the absence of a history of cardiovascular disease [71]. This is a presentation of fulminant myocarditis, a condition characterized by sudden and severe cardiac inflammation, which may lead to arrythmias, severe heart failure or death [72, 73]. Physicians should be vigilant with patients who present with hypotension, ECG changes (e.g., ventricular tachycardia, bradyarrythmias, ST depression) or clinical signs of heart failure such as peripheral edema [73].

## Diagnosing COVID-19 myocarditis

C-reactive protein (CRP), lactate dehydrogenase (LDH) and white cell count (WCC) have been shown to be raised in patients with COVID-19 myocarditis [68–70]. These blood tests are markers of infection and, thus, are non-specific to myocarditis. Raised cardiac enzymes (e.g., troponin) and N-terminal pro-B-type natriuretic peptide (NT-pro-BNP) have also been noted in COVID-19 myocarditis [44, 70]. Therefore, it is a good idea to take baseline levels of troponin I/T and NT-pro-BNP on admission of a patient with COVID-19 allowing a trend of these tests to be established throughout the patients stay [28]. However, some patients with COVID-19 myocarditis may not have a raised troponin, meaning a normal troponin does not rule out myocarditis [22, 74]. In fact, the sensitivity for elevated troponin I levels in myocarditis is 34% [75]. Electrocardiogram (ECG) changes can also be seen with patients who develop myocarditis. Most changes are non-specific and can include sinus tachycardia (the most common change), ST segment elevation/depression, T wave inversions, tachy/bradyarrhythmia and QT prolongation [44, 70]. Therefore, ECG changes are not diagnostic of myocarditis, but they may be helpful as a tool to assess for possible myocardial damage or the presence of arrythmias, indicating severity of disease. Echocardiography may also be used; however, it also shows non-specific changes in myocarditis, including reduced ejection fraction, pericardial effusion and hypokinesis throughout the heart wall [74, 76]. Echocardiography can be useful to rule out other causes of heart failure, such as valvular or congenital causes [74].

cMRI has a high sensitivity for diagnosing myocarditis and is therefore the best noninvasive test [77]. The Lake Louis criteria should be used when interpreting cMRI images [78]. This criterion uses a combination of T2-weighted images, early gadolinium enhancement and late gadolinium enhancement to detect myocardial edema, hyperemia and myocardial necrosis and fibrosis, respectively [78–80]. cMRI has shortcomings as it is not possible to distinguish whether the inflammation is caused from an autoimmune response to the virus or from viral infection of the myocardium [78]. Furthermore, when used in patients presenting with severe myocarditis causing cardiogenic shock or hemodynamic instability application of cMRI can be limited as these patients may be mechanically ventilated or have tachyarrhythmias [77, 81]. In these situations, it may be preferred to use EMB which is considered the gold standard test to confirm the presence of myocarditis as it can determine the nature of inflammation [77]. For example, samples from the biopsy can be sent for immunohistology and genomic analysis, which can confirm the diagnosis of COVID-19-induced myocarditis through the presence of SARS-CoV-2 RNA [82]. EMB samples were previously interpreted using the Dallas criteria which described myocarditis as myocyte necrosis or damage associated with inflammatory infiltrates [83]. The reliability of the Dallas criteria is questionable as it has been shown to not apply to 50% of virus positive cases [29]. EMB has further drawbacks including risk of infection and sampling errors due to the patchy inflammation seen in myocarditis [74]. An immunohistochemistry criteria has been added to the Dallas criteria to make it more reliable [29]. This criterion defines myocarditis as the presence of leukocytes ≥ 14/mm^2^ with monocytes ≤ 4/mm^2^ and CD3 + cells ≥ 7/mm^2^ alongside evidence of non-ischemic necrosis under histology [29]. Using this criteria may increase the sensitivity of cMRI in diagnosing COVID-19 myocarditis.

During the COVID-19 pandemic, the availability of scans, such as cMRI, has been greatly reduced, while due to the risk of infection EMB is likely avoided on patients with COVID-19 [70, 84]. Therefore, physicians may need to use a combination of blood tests, ECGs, echocardiograms and a high clinical suspicion for myocarditis to reach a diagnosis if hospitals are under pressure from COVID-19.

## Managing COVID-19 myocarditis

Treating myocarditis involves the management of both the myocardial inflammation and the complications that may arise from it. Intravenous immunoglobulins (IVIG) have been studied for their efficacy in treating viral myocarditis. IgG, IgA and IgM immunoglobulins have anti-inflammatory effects, while neutralizing and facilitating the clearance of pathogens from the myocardium [85]. Maisch et al. demonstrated that immunoglobulin therapy for biopsy proven cytomegalovirus myocarditis showed favorable outcomes with a reduction in inflammatory and viral levels [86]. However, in cases of suspected myocarditis with no biopsy proof of viral infection, use of immunoglobin therapy showed inconsistent results [86]. Hu et al. used a combination of glucocorticoid and immunoglobulin treatment to successfully treat COVID-19 myocarditis [87]. A meta-analysis revealed that the use of IVIG to treat acute myocarditis significantly reduced mortality, while improving left ventricular ejection fraction [88]. Moreover, the effect of IVIG was even more noticeable in patients with fulminant myocarditis where it showed to significantly increase survival rates of this life-threatening condition [88]. The evidence for use of corticosteroids to treat COVID-19 myocarditis is not as clear. The use of the corticosteroid, prednisolone, may be effective in treating viral myocarditis in the absence of viral replication [89]. It is thought that the use of immunocompromising medication, such as corticosteroids, may worsen acute myocarditis where viral replication is present [90]. A systematic review by Sawalha et al. revealed the use of corticosteroids showed improved outcomes among patients with COVID-19 myocarditis [76]. This comes with the caveat that the review consisted of 14 case reports, and therefore, the evidence lacks in reliability. On the other hand, other studies show the use of corticosteroid therapy does not reduce mortality in patients with viral myocarditis [91]. Tocilizumab, which is an anti-IL-6 receptor monoclonal antibody, was trialed with the combination of the anti-viral, favipiravir, to treat COVID-19 patients who had developed cytokine storm [92]. The trial found that the combination of Tocilizumab and favipiravir significantly reduced inflammation caused by cytokine storm [92]. As COVID-19 myocarditis may be exacerbated by cytokine storm, the use of this combination therapy may provide positive outcomes [28]. Although there is evidence to show the efficacy of IVIG in the treatment of viral myocarditis, more research is needed to assess the effects on COVID-19 myocarditis specifically. Dexamethasone, a corticosteroid, is currently used in the management of COVID-19. Therefore, research into assessing the efficacy of Dexamethasone may reveal if current treatment is sufficient or if patients who develop COVID-19 myocarditis require extra anti-viral/anti-inflammatory treatment.

Patients who present with cardiogenic shock due to fulminant myocarditis need further management. For patients with cardiogenic shock, the use of inotropic agents, such as dobutamine, and mechanical support, including intra-aortic balloon pumps or Impella systems, can be used to maintain blood pressure [74, 77]. The presence of tachyarrhythmias can be treated with intravenous amiodarone hydrochloride, or, if the patient is unstable or unresponsive to pharmacological intervention, direct current cardioversion or pacing may be used [93]. Bradyarrhythmias which may occur can be treated by intravenous atropine or, if required, transcutaneous pacing can be commenced [93].

## Conclusions

COVID-19 myocarditis is a significant complication of SARS-CoV-2 infection which can worsen the prognosis for patients. While some cases may be insignificant or asymptomatic, it is likely clinicians will come across cases which are more severe and require prompt treatment. Therefore, it is imperative to recognize how to diagnose and treat this condition. If possible, it is a good idea to carry out serial troponins and ECGs to monitor for any development of myocarditis or other myocardial injury. As the symptoms for myocarditis can be non-specific and can overlap with the respiratory symptoms of COVID-19, it may be hard to diagnose this condition. It is important to have a low threshold to work up a patient, as initial tests are relatively inexpensive. Further testing should be used for patients with evidence of myocardial injury on initial work up, these include echocardiography, cMRIs and EMB. Clinicians should be vigilant for any signs of heart failure or arrhythmias, as these could be the life-threatening signs of fulminant myocarditis. While no definitive treatment for COVID-19 myocarditis has been published, the combination of IVIG and corticosteroids shows promise to reduce mortality, particularly in the case of fulminant myocarditis.

## Data Availability

Not applicable.

## Abbreviations

COVID-19: Coronavirus disease 2019
SARS-CoV-2: Severe acute respiratory syndrome coronavirus 2
S: Spike
ACE: Angiotensin-converting enzyme
TMPRSS2: Type 2 transmembrane serine protease
IL: Interleukin
EMB: Endomyocardial biopsy
cMRI: Cardiac magnetic resonance imagining
BAME: Black, Asian and minority ethnic
CRP: C-reactive protein
LDH: Lactate dehydrogenase
WCC: White cell count
NT-pro-BNP: N-terminal pro-B-type natriuretic peptide
ECG: Electrocardiogram
IVIG: Intravenous immunoglobulins
